# MECHANISMS OF ATTENTIONAL CONTROL AND SUPPRESSION IN AGING

**DOI:** 10.1093/geroni/igad104.2625

**Published:** 2023-12-21

**Authors:** Josh Senior, Andy Kim, Mara Mather

**Affiliations:** University of Southern California, Los Angeles, California, United States; University of Southern California, Los Angeles, California, United States; University of Southern California, Los Angeles, California, United States

## Abstract

Attentional control plays a crucial role in our daily lives by allowing our brains to selectively process relevant information while filtering out task-irrelevant distractions. Due to aging, older adults have demonstrated a heightened propensity to be more distractible. This decreased ability to regulate attention has been attributed to an inability to inhibit task-irrelevant salient stimuli. However, the specific mechanisms that underlie this age-related alteration remain unclear. In this study, we utilized a visual search paradigm that directly measures mechanisms of attentional suppression to test our hypothesis that older adults demonstrate reduced capabilities to suppress task-irrelevant information. Young adults (aged 18-35) and older adults (aged 50-80) were tasked to utilize goal-directed attentional control by fixating at a specific target shape. Critically, in some trials, individuals were exposed to a salient color shape that acted as a singleton distractor. While young adults show the ability to suppress attentional allocation to this salient distractor in this paradigm, we hypothesized that older adults would demonstrate attentional capture by this salient distractor. Our findings revealed that older adults made significantly fewer first-fixations towards the target, demonstrating a marked impairment with goal-directed attentional control. Surprisingly, older adults still made fewer fixations toward the distractor in comparison to non-targets, demonstrating that older adults sustained the ability to suppress irrelevant distractors. These findings raise important questions on the mechanisms that underlie increased distractibility in aging and oculomotor attentional control.

